# PAEs Derivatives’ Design for Insulation: Integrated In-Silico Methods, Functional Assessment and Environmentally Friendly Molecular Modification

**DOI:** 10.3390/ijerph19063232

**Published:** 2022-03-09

**Authors:** Haigang Zhang, Chengji Zhao, Hui Na

**Affiliations:** Alan G. MacDiarmid Institute, Jilin University, Changchun 130012, China; zhaochengji@jlu.edu.cn (C.Z.); huina@jlu.edu.cn (H.N.)

**Keywords:** plasticiser, phthalic acid esters, composite index method, insulation, 3D-QSAR

## Abstract

As a common substance in production and life, phthalic acid esters (PAEs), the main component of plastics, have brought more and more serious problems to the environment. This study normalized the insulation, toxicity, and bioconcentration data of 13 PAEs to eliminate the dimensional coefficients of each index, and then used the comprehensive index method to calculate the comprehensive effect value of PAEs with three properties. The comprehensive effect value was used as the data source to construct the 3D-QSAR model of PAE molecular comprehensive effect. The DAP was selected as the target molecule, the distribution of each force field in the three-dimensional equipotential map was analyzed, and 30 molecular modification schemes were created. The constructed single-effect models of insulation, toxicity, and bioconcentration of PAEs and the scoring function module of DS software were used to evaluate the stability and environmental friendliness of PAE derivative molecules. Four PAE derivatives were screened for increased comprehensive effects, enhanced insulation, and reduced toxicity and bioconcentration. By calculating the binding energy of the target molecule and the derivative molecule with the degrading enzyme under different applied electric fields, it was found that the binding energy of DAP-1-NO2-2-CH2C6H5 decreases more than DAP does when there is an applied electric field, indicating that the degradation ability of degrading enzymes on PAE derivative molecules is reduced, which indirectly proves that the insulation is enhanced. The innovation of this paper lies in the insulation, toxicity, and bioenrichment data of PAEs being processed by mathematical method for the first time, and PAEs with high insulation, low toxicity, and low bioconcentration were designed by building a comprehensive model.

## 1. Introduction

Plastics are polymeric compounds formed through polyaddition or polycondensation and present many advantageous properties such as light weight, stability, high insulation, impact resistance, and abrasion resistance [1]. With the rapid development of society and substantial improvement in the living standards of people, plastic products have been widely used and have gradually become an indispensable part of daily life, work, and travel [2,3]. For different occasions and environments, plastic products should meet certain performance indicators [4,5]. For example, in cable materials, in addition to meeting the requirements of mechanical properties, heat resistance, and abrasion resistance, plastic products should show good electrical insulation [6]. Studies have shown that adding plasticizers to plastic products can greatly reduce the insulation of plastics [7]. Nagy [8] et al. reported the effect of DOP on PVC dielectric properties, indicating that the increase of plasticizer is proportional to the insulation of PVC.

Phthalic acid esters (PAEs) are also called phthalates and are usually used as plasticizers in plastic products to enhance their ductility and flexibility [9]. PAEs are difficult to dissolve in water and are easily soluble in organic solvents [10]. Thus, PAEs are difficult to combine with high polymer chains, but they can be combined with these chains through van der Waals forces or hydrogen bonding, which facilitates them to break from plastic products and enter atmosphere, water, soil, and other environmental media [11,12]. Then, these PAEs can accumulate in organisms through various means such as absorption, ingestion, respiration, and contact [13]. Most PAEs in the air emerge from the production and incineration of plastic products, spraying of coatings, and volatilization of plasticizers in agricultural plastic films; these PAEs have been detected in the atmosphere worldwide [14]. Although the natural complete degradation of plastic is difficult, it can be decomposed into microplastics through processes including sun exposure, weathering, and abrasion, which leads to several adverse effects, such as PAE accumulation and enrichment in organisms through food chains, which endangers organism health and the natural environment [15]. Dubaish et al. [16] reported the frequent appearance of suspended microplastics and black carbon particles in the coastal waters of the southern North Sea. Zhou [17] found that in the Bohai Sea, many benthic organisms, such as larval cockles, portunus snails, and red snails, exhibited microplastic accumulation. Zhao et al. [18] detected 39 volatile and semivolatile organic compounds in the water samples of Taihu Lake, and the content of PAEs was the highest. Wang et al. [19] detected a high concentration of PAEs in greenhouse soil covered with plastic film, and PAEs could accumulate in vegetables grown in this soil.

In recent years, with the increase in awareness regarding the pollution from environmental endocrine disruptors, environmental and health problems resulting from phthalate substances have become highly prominent. Countries such as China, the European Union, Japan, and the United States have all listed PAEs as environmentally superior pollutants [20]. Studies have shown that PAEs can enter organisms through various means, such as air, food, and body surface contact. Moreover, through the food chain, PAEs accumulate in the body, thereby producing multiple toxic effects, such as estrogenic/antiestrogenic effects and effects on androids/antiandrogens and thyroid hormone/antithyroid hormone [21]. Saillenfait [22] reported that exposure to diisooctyl phthalate during pregnancy can cause various reproductive system abnormalities, such as hypospadias and insufficient testis production, and can show an antiandrogen activity, thus disrupting normal male reproductive development. Shi et al. [23] investigated the urine samples of children aged 7–14 years and found a significant correlation between phthalate metabolite concentrations and puberty. Wang et al. [24] found that phthalate compounds can be detected in food containers, and their food simulant migration test proved that when PAEs migrate towards food, their content exceeds the standard limit, which causes toxic effects on the human body.

In view of the consistent performance of the insulation, bioconcentration, and toxicity of the PAEs in natural environments and organisms, the exploration of environmentally friendly phthalate derivatives with high insulation, low bioconcentration, and low toxicity is critical. This paper first normalizes the activity data of the insulation, bioconcentration, and toxicity of PAE molecules, uses the improved comprehensive index method to obtain the comprehensive effect value of PAEs with three properties, and uses this effect value as the data source to construct the 3D-QSAR comprehensive effect model of PAE molecules. Then, using DAP as the target molecule for molecular modification, 30 PAE derivative molecules with enhanced insulation and decreased toxicity, and bioconcentration are designed and screened. Finally, molecular dynamics is used to analyze the differences in the activity and mechanism of PAEs and their derivatives.

## 2. Materials and Methods

### 2.1. Data Source

In this paper, the comprehensive model and single model for PAEs were constructed by using the insulativity, toxicity, and bioconcentration parameters of 13 PAEs as indexes. Among them, the insulativity data of PAEs, which was expressed by their permittivity, was obtained by molecular dynamics. The higher the permittivity, the stronger the insulation [25]. The toxicity data of PAEs were expressed by median lethal concentration of 50% (*LC*_50_) of PAEs to fish, cited from Estimation Program Interface (EPI) Suite database [26]. The lower the *LC*_50_ value, the stronger the PAE toxicity. The bioconcentration data (bioconcentration factor, log *BCF*) of PAEs were predicted using the EPI database [27]. The greater the log *BCF* value, the stronger the PAE enrichment. Table 1 shows each parameter.

### 2.2. The Comprehensive Data of Insulativity, Toxicity, and Bioconcentration of PAEs Processed by Comprehensive Index Method

The comprehensive index method is a comprehensive evaluation method used to transform multiple variable indicators into a comprehensive index to reflect the overall situation of evaluation subjects [28]. This method can be used not only to reflect the direction and degree of the overall change in complex economic phenomena but also to quantitatively explain actual economic effects caused by the change in phenomena. Especially in a complex model of multiple indicators, this method can consider not only the impact of indicators but also the interrelationship among them [29]. The relevant operation steps are as follows: first, normalization is performed to eliminate the dimensional coefficient between each index [30]; then, the mean method is used to determine the weight for each index; finally, the comprehensive index method is employed to integrate the given weights of indicators into the comprehensive index.

In this study, three property parameters of PAEs were arranged according to the numerical size to separately screen the maximum and minimum values of each indicator. According to the properties of indicator parameters, the positive or negative index formula was selected to normalize the parameters for dimension elimination. Then, the weight of each index was determined as 25:23:52 by using the mean method. Finally, the comprehensive index method was used to process the three weighted indicator parameters to integrate these parameters into the comprehensive effect values of 13 PAEs, which can effectively contain the PAE insulativity, toxicity, and bioconcentration information according to a certain transformation relationship. The specific calculation steps and formulas are as follows [30]:

(1) Normalization

(1) $$For\;the\;positive\;indicator\;of\;PAEs:\;q_{ij}=\frac{v_{ij}-min\;\left(v_{ij}\right)}{\max\;\left(v_{ij}\right)-min\;\left(v_{ij}\right)}$$

(2) $$For\;the\;negative\;indicator\;of\;PAEs:\;q_{ij}=\frac{\max\;\left(v_{ij}\right)-v_{ij}}{\max\;\left(v_{ij}\right)-min\;\left(v_{ij}\right)}$$

(2) Comprehensive index method

(3) $$I=\overset{m}{\underset{i\;=\;1}{\sum }}w_{i}y_{i}$$

### 2.3. Construction of Comprehensive Model for PAEs’ Insulativity, Toxicity, and Bioconcentration

Using the Minimize module in Sybyl-X 2.0 software, the three-dimensional structures of PAEs were optimized using the Powell conjugate gradient method to obtain the low-energy conformation of each molecule, with selected Gasterger–Hückle charge and Tripos molecular force field. The energy convergence standard and iteration number were set to 0.005 kcal mol^−1^ and 10,000, respectively [31].

The PAE molecule with a high activity was selected as the template, the Align Database module was used to superpose the skeletons of multiple PAE compounds, and the molecular parameters of CoMFA and CoMSIA fields were calculated. Model construction involved the following steps: First, the molecular structures of PAEs available in the training set were imported into the training table, and CoMSIA field parameters were calculated using the Autofill module. Among the CoMSIA fields, three-dimensional hydrophobic, electrostatic, hydrogen bond donor, and hydrogen bond acceptor fields were selected as the molecular field. Relative permittivity was selected by referring to the distance, and the threshold was set to 125.4 kJ/mol. The remaining parameters followed the default system settings. Subsequently, when partial least squares method was used to analyze the conformational relationship between molecular structure and biological activity [32], leave-one-out method was selected for cross-validation of training set compounds [33] to calculate the cross-validation coefficient (q^2^ > 0.5) and the optimal principal component number (n). No validation regression analysis was performed to obtain the non-cross validation coefficients (r^2^ > 0.6), standard error of estimate (SEE), and test value (F) [34]. Finally, the test set constituting the remaining molecules was used to verify the constructed model. The constructed model exhibited high stability and universality when the calculated cross-validation coefficients (r^2^_pred_ > 0.6) and standard error of predict (SEP) met the conditions [35,36].

In this paper, 10 PAE molecules were randomly selected from 13 PAEs (including template molecules) with comprehensive effect values as training sets in a ratio of about 3:1, and a 3D-QSAR comprehensive model with PAEs’ insulation, toxicity, and bioconcentration was constructed. The remaining 4 PAEs (including template molecules) were used as the test set for the internal verification of the constructed comprehensive model.

## 3. Results and Discussion

### 3.1. Calculation of the Comprehensive Effect Value of PAEs with Insulation, Toxicity, and Bioconcentration

The insulation, toxicity, and bioconcentration data of PAE molecules processed using the normalization method were weighted (25:23:52), and then, the comprehensive effect value of 13 PAEs was calculated by employing the synthetic index method (Table 2).

### 3.2. Construction and Evaluation of the CoMSIA Model of PAEs’ Comprehensive Effect

#### 3.2.1. Construction of CoMSIA Model of PAEs’ Molecular Comprehensive Effect

The data presented in Table 2 were used to construct the CoMSIA model of PAEs’ comprehensive effect with insulation, toxicity, and bioconcentration. Then, the three-dimensional contour maps were used to analyze the proportion of the target molecule (DAP) in each force field. The model evaluation parameters are presented in Table 3.

The optimal principal component (n) of the comprehensive effect CoMSIA model is 6, and the cross-validation coefficient (q^2^) is 0.747 (>0.5) (Table 3), indicating that the prediction ability of the developed model has a certain degree of credibility. The non-cross validation coefficient (R^2^) is 0.929 (>0.9), the standard deviation (SEE) is 0.100, and the F value is 26.209, indicating that the model exhibits good fitting ability and robustness [37,38]. The model has certain reliability and universality in the prediction of the PAEs’ comprehensive effect value with insulation, toxicity, and bioconcentration. Simultaneously, the CoMSIA model was used to observe the contribution rate of each force field. The contribution rates of DAP to the steric, electrostatic, hydrophobic, hydrogen bond donor, and hydrogen bond acceptor fields are 31.5%, 13.3%, 30.9%, 0.00%, and 12.3%, respectively. This finding showed that the comprehensive effect CoMSIA model can have a relatively large effect on the steric space, electrical distribution, hydrophobicity, and hydrogen bond acceptor of PAE molecules, while the influence of the hydrogen bond donor field on PAE molecules can be ignored.

#### 3.2.2. Analysis of Three-Dimensional Contour Maps of PAEs’ Comprehensive Effect Model

**Figure 1:**
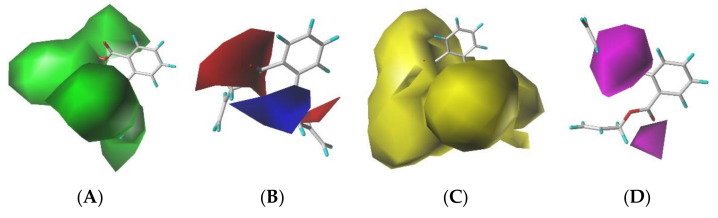
Three-dimensional contour maps of (**A**) steric, (**B**) electrostatic, (**C**) hydrophobic, and (**D**) hydrogen bond acceptor fields.

The DAP molecule was selected as the target molecule, the 3D-QSAR model of the combined effect with insulation, toxicity, and bioconcentration was imported, and the three-dimensional contour maps were analyzed by observing and comparing the color blocks of the DAP molecule in different force fields to obtain the factors affecting the DAP molecule activity under different force fields [39]. The three-dimensional contour map can visually and effectively show the interaction between small molecules and their receptors. It provides a certain basis for molecular modification, and the closer the molecule is to the color patch area of the contour map, the more beneficial the enhancement of molecular activity. There are four force fields in the CoMSIA model that can have different effects on the molecule activity in the field (Figure 1). In the steric field, the green region indicates that the introduction of bulky groups in this area can enhance the overall molecule activity. In the electrostatic field, the blue and red regions indicate that the introduction of positively and negatively charged groups, respectively, in this area improves the molecule activity. In the hydrophobic field, the yellow region indicates that the introduction of hydrophobic groups enhances the compound activity. In the hydrogen bond acceptor field, the purple-red region indicates that the increase in the hydrogen bond acceptor is conducive to molecule activity enhancement. Combined with the contribution rate of molecular field and the three-dimensional contour maps, this study ignored the influence of electrostatic field, hydrogen bond, and hydrogen receptor field when there was molecular modification of the target molecule, and only considered the influence of steric field and hy-drophobic field with the highest contribution rate in the molecular force field. In Figure 1A, it can be observed that the H atom attached to C-2 and the vicinity of the C-2′ atom of the DAP molecule are all green, and the yellow area in Figure 1C basically covers the side chain on the benzene ring of the DAP molecule. This indicates that the introduction of bulky or hydrophobic groups at the sites covered by the side chains of DAP molecules can improve the PAEs’ comprehensive effect values of insulation, toxicity, and bioconcentration.

### 3.3. Modification and Evaluation of DAP Derivatives Based on the CoMSIA Model

**Figure 2:**
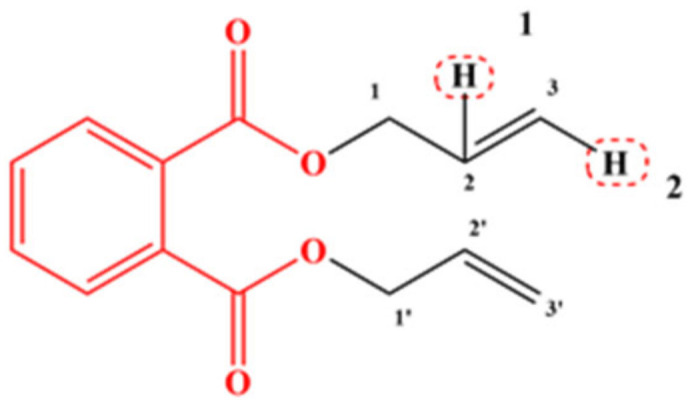
Site map of the molecular modification of DAP.

Comprehensively considering the three-dimensional equipotential map information and the hydrophobic fields, the modification sites shown in Figure 2 were selected to replace and modify the DAP molecule. According to the molecular modification site map, 13 groups with large volume and hydrophobic characteristics, including –F, –CL, Br, –SH, –CH_3_, –CH_2_CH_3_, –CH_2_CH_2_CH_3_, –C_6_H_5_, –CH_2_C_6_H_5_, –NO_2_, –CH_2_NO_2_, and –OCH_3_, were selected to modify the DAP at single site and dual site. A total of 56 DAP derivative molecules were designed, among which 27 exhibited single-site modification and 29 showed dual-site modification. The comprehensive effect and single effect of 56 designed PAE derivatives were predicted using the constructed PAEs’ comprehensive effect model with insulation, toxicity, and bioconcentration and the single-effect models of insulation, toxicity, and bioconcentration, respectively. Consequently, a total of 30 DAP derivative molecules with higher comprehensive effect of insulation, toxicity, and bioconcentration than that of the target molecule (DAP), no reduction in insulation, and decreased toxicity and bioconcentration were screened (Table 4). This result indicated that the designed comprehensive effect model of PAE derivatives can effectively combine the molecular information of insulation, toxicity, and bioconcentration and provide a reliable basis for the modification of PAEs. The specific prediction results are presented in Table 4.

### 3.4. Mechanism Analysis

#### 3.4.1. Mechanism Analysis of Improvement of Molecular Toxicity and Bioconcentration of DAP Derivatives Based on Molecular Docking

Discovery Studio (DS) software is used for molecular modelling and simulation and presents a very wide range of applications in molecular design. The scoring function value for DS can be used to characterize the binding strength between the ligand and receptor [40]. Therefore, insulative, toxic, and bioenriching receptors were selected, and the pharmacophore module of DS was used for the molecular docking of the target molecule and 30 PAE derivatives. The insulative receptor was derived from the carboxylic acid esters of thermophilicbacterium bacillus [41] (PDB ID: 5UOH), the toxicity receptor was deoxytocin synthase (PDB ID: 6XXK), and the bioconcentration receptor was obtained from cytochrome P-450 [42] (PDB ID: 4R20). The scoring function value shows that among the 30 PAE derivatives, 4 PAE derivatives have lower toxicity and biological concentration than that of the target molecule without reducing the insulation. Compared with the DAP molecule, the insulation, toxicity, and bioconcentration of DAP-1-NO_2_-2-C_6_H_5_ increased by 21.23%, decreased by 3.73%, and decreased by 10.72%, respectively. This finding showed that the designed derivative molecules have good parameter performance (high insulation, low toxicity, and low bioconcentration), and the PAEs’ comprehensive effect model can simultaneously consider the information of insulation, toxicity, and bioconcentration. The specific analysis results are shown in Table 5.

#### 3.4.2. Mechanism Analysis of Insulation Improvement of PAE Derivatives Based on Molecular Dynamics

In order to further explore the related mechanism and effect of the improvement of the performance of PAE derivative molecules compared with target molecules, in this paper, by means of molecular dynamics simulation, carboxylesterase [43] (PDB ID: 5UOH) was used as PAEs’ degradation enzyme, and DAP and its derivatives were respectively docked using SYBYL-2.0 X software to obtain the corresponding protein ligand complex. Then, GROMACs molecular dynamics software and simulation method of g-MMPBSA were used to simulate the binding free energy (ΔG) of DAP and its derivatives with degradation enzyme under different applied electric field conditions (Table 6) [44]. Among them, the intermolecular binding free energy can quantitatively indicate the strength of the intermolecular interaction to a certain extent [45]. The negative value indicates that the receptor and ligand can effectively bind and produce corresponding effects, and the smaller the ΔG of PAEs and degrading enzymes, the stronger the degradation of PAEs by the enzymes. Studies have found that the applied electric field can promote cycloalkane degradation [46]. Tiehm et al. [47] reported that the applied electric field can stimulate the ability of noncharged compounds to degrade pollutants.

Under the conditions of no applied electric field (0 V) and applied electric field (5 V), the binding free energies of DAP, its three derivatives, and degradation enzyme receptor (5UOH) are <0, which indicates that this enzyme has a certain degradation effect on PAEs and their derivatives. In addition to DAP-1-NO_2_-2-CH_2_C_6_H_5_, the binding free energy values of the remaining two derivatives (DAP-1-NO_2_-2-CH_2_CH_2_CH_3_ and DAP-1-NO_2_-2-OCH_3_) decreased to different degrees (ΔG_0V_ = −14.71%, 70.10%; ΔG_5V_ = −110.79%, −290.82%) compared with the target molecule (DAP), indicating that the designed derivatives have better stability and environmental friendliness. Moreover, the comparison of the free binding energy changes of the target molecule and the derivative molecules of PAEs in the absence and presence of the applied electric field showed that the free binding energy increases with the applied electric field. The binding free energy of DAP with applied electric field was 44.932 kJ/mol higher than that without applied electric field. The binding free energy of DAP-1-NO_2_-2-CH_2_C_6_H_5_ with the applied electric field increased by 69.630 kJ/mol compared to without the applied electric field. The results show that the degradation ability of 5UOH to the derivative molecule is reduced, that is, the insulation of DAP-1-NO_2_-2-CH_2_C_6_H_5_ molecule is enhanced, and this molecule can be used as the preferred derivative molecule with enhanced PAE insulation.

## 4. Conclusions

The 3D-QSAR model of PAEs’ comprehensive effects with insulation, toxicity, and bioconcentration was constructed by using the comprehensive index method for the first time. A total of 30 PAE derivatives with enhanced insulation and reduced toxicity and bioconcentration were designed and screened by using the three-dimensional equipotential map of PAEs’ multi-effect model and the models of comprehensive effect and single effect of insulation, toxicity, and bioconcentration. Four PAE derivative molecules with high insulation, low toxicity, and low bioconcentration were screened by comparing the scoring function values of insulation, toxicity, and bioconcentration of PAEs and their derivatives before and after modification. Analysis of molecular dynamics simulations showed that the increase in the free binding energy of DAP-1-NO_2_-2-CH_2_C_6_H_5_ molecule was higher than that in the DAP molecule when the electric field was applied, which can explain the mechanism of enhanced insulation of the designed PAE derivative molecule. This paper provides a theoretical basis for the design of novel PAE plasticizers with high insulation, low toxicity, and low bioaccumulation.

## Figures and Tables

**Table 1:** Parameters of insulativity, toxicity, and bioconcentration of 13 PAEs.

PAEs	Permittivity	Toxicity	Bioconcentration
	(F/m)	*LC*_50_ to Fish (mg/L)	log*BCF*
DAP	1.00184	5.323	1.798
DEP	1.00109	12.471	1.264
DHP	1.01600	0.095	2.793
DIBP	1.00560	1.356	2.379
DIHP	1.00023	0.028	3.255
DIHXP	1.00115	0.116	3.908
DIPP	1.00016	0.398	3.260
DIPRP	1.00005	4.568	1.534
DMP	1.00310	40.822	0.402
DPP	1.00459	0.327	2.988
DPRP	1.00214	3.749	2.001
DTDP	1.04921	1.000 × 10^−5^	1.825
DMEP	1.00087	124.130	1.330

**Table 2:** Comprehensive effect value (I) of PAEs’ molecular insulation, toxicity, and bioconcentration.

PAEs	Permittivity (F/m)	Toxicity Fish LC_50_ (µg/L)	Predictive Value of Bioconcentration	I
^a^ DAP	0.81	42.882	0.60	0.52
^a^ DEP	1.00	100.467	0.75	0.66
^a^ DHP	0.67	0.765	0.32	0.33
^a^ DIBP	0.82	10.924	0.44	0.44
^b^ DIHP	0.84	0.225	0.19	0.31
^a^ DIHXP	0.96	0.934	0.18	0.33
^b^ DIPP	0.96	3.206	0.18	0.33
^a^ DIPRP	0.79	36.800	0.68	0.56
^a, b^ DMP	1.00	328.865	1.00	0.85
^a^ DPP	0.65	2.634	0.26	0.30
^a^ DPRP	0.91	30.202	0.54	0.52
^a^ DTDP	0.75	0.000	0.59	0.49
^b^ DMEP	0.96	1000	0.74	0.85

^a^ Training set; ^b^ Test set.

**Table 3:** PAEs’ molecular comprehensive effects CoMSIA model evaluation parameters and molecular field contribution rate statistics.

Model	*n*	q^2^	r^2^	SEE	F	S	E	H	D	A
CoMSIA	6	0.747	0.929	0.100	26.209	31.5%	13.3%	30.9%	0.00%	12.3%

S: steric; E: electrostatic; H: hydrophobic; D: donor; A: acceptor.

**Table 4:** The predicted value and percentage change of PAE derivatives based on the 3D-QSAR model of combined and single effects.

PAEs and Their Derivatives	Predicted Value of Comprehensive Model	Change Percentage (%)	Predicted Value of Insulation	Change Percentage (%)	Predictive Value of Toxicity	Change Percentage (%)	Predicted Value of Enrichment	Change Percentage (%)
DAP	0.61	-	1.002	-	0.73	-	1.80	-
DAP-1-Br	0.65	6.23	1.002	0.00	0.87	18.49	1.64	−8.62
DAP-1-CH_2_C_6_H_5_	0.71	17.05	1.003	0.10	0.92	26.30	1.47	−18.19
DAP-1-CH_2_NO_2_	0.87	43.11	1.002	0.00	2.28	212.47	1.01	−43.99
DAP-1-F	0.72	18.03	1.002	0.00	1.11	52.05	1.35	−24.69
DAP-1-NO_2_	0.73	19.02	1.002	0.00	2.01	174.79	1.48	−17.58
DAP-1-SH	0.73	19.18	1.002	0.00	1.13	54.52	1.36	−24.36
DAP-2-Br	0.75	22.62	1.004	0.20	1.22	66.71	1.28	−28.92
DAP-2-CH_2_NO_2_	0.97	59.34	1.004	0.20	2.27	210.41	0.16	−90.99
DAP-2-CL	0.67	9.51	1.002	0.00	0.81	10.68	1.78	−0.78
DAP-2-COOCH_3_	0.73	19.34	1.004	0.20	0.83	14.11	1.21	−32.93
DAP-2-F	0.73	19.84	1.002	0.00	1.00	36.85	1.39	−22.69
DAP-2-NO_2_	0.97	58.85	1.003	0.10	1.94	165.89	0.18	−89.82
DAP-2-SH	0.69	13.77	1.002	0.00	0.94	28.63	1.60	−10.79
DAP-1-NO_2_-2-C_6_H_5_	0.77	26.89	1.003	0.10	1.06	44.66	1.35	−25.08
DAP-1-NO_2_-2-CH_2_C_6_H_5_	0.85	39.34	1.003	0.10	1.06	127.81	1.18	−34.26
	0.79	29.51	1.005	0.30	0.91	24.52	0.83	−53.78
DAP-1-NO_2_-2-CH_2_CH_3_	0.82	34.43	1.004	0.20	1.88	157.95	1.48	−17.58
DAP-1-NO_2_-2-CH_2_NO_2_	1.10	79.84	1.004	0.20	2.55	249.45	1.32	−26.36
DAP-1-NO_2_-2-CH_3_	0.79	30.16	1.005	0.30	0.92	26.30	0.83	−53.84
DAP-1-NO_2_-2-CH=CH_2_	0.89	45.57	1.005	0.30	2.03	177.81	0.70	−61.29
DAP-1-NO_2_-2-CL	0.87	42.46	1.003	0.10	1.23	68.08	1.29	−28.31
DAP-1-NO_2_-2-COOCH_3_	1.07	74.92	1.002	0.00	0.93	27.26	1.35	−25.08
DAP-1-NO_2_-2-F	1.05	72.46	1.002	0.00	2.04	178.90	0.72	−59.73
DAP-1-NO_2_-2-NO_2_	1.31	114.43	1.003	0.10	3.20	338.22	0.76	−57.68
DAP-1-NO_2_-2-OCH_3_	0.84	37.21	1.006	0.40	1.83	150.96	1.40	−22.08
DAP-2-CH=CH_2_-1-CH_2_NO_2_	0.83	36.39	1.003	0.10	0.95	30.41	0.90	−50.06
DAP-2-CH=CH_2_-1-CH_3_	0.69	12.46	1.004	0.20	0.90	23.70	1.37	−24.03
DAP-2-CH=CH_2_-1-NO_2_	0.88	44.26	1.006	0.40	1.67	128.49	1.17	−35.15
DAP-2-CH=CH_2_-1-OCH_3_	0.74	21.97	1.003	0.10	0.94	28.08	1.39	−22.53
DAP-2-CH=CH_2_-1-SH	0.76	24.92	1.002	0.00	0.84	14.38	1.46	−18.85

**Table 5:** Scoring function value and percentage change of PAE derivatives based on DS software.

Molecular	Scoring Function Value of Insulation	Percentage Change (%)	Scoring Function Value of Toxicity	Percentage Change (%)	Scoring Function Value of Bioconcentration	Percentage Change (%)
DAP	69.56	-	70.49	-	65.46	-
DAP-1-NO_2_-2-C_6_H_5_	84.33	21.23	73.12	3.73	58.44	−10.72
DAP-1-NO_2_-2-CH_2_C_6_H_5_	100.31	44.20	88.95	26.19	64.83	−0.95
DAP-1-NO_2_-2-CH_2_CH_2_CH_3_	79.05	13.65	80.06	13.58	62.49	−4.54
DAP-1-NO_2_-2-OCH_3_	87.28	25.47	77.89	10.49	62.56	−4.43

**Table 6:** Binding free energy of PAE derivatives and percentage change under different applied electric fields.

Molecular	ΔG_0V_ (kJ/mol)	Percentage Change (%)	ΔG_5V_ (kJ/mol)	Percentage Change (%)
DAP	−80.027	-	−35.095	-
DAP-1-NO_2_-2-CH_2_C_6_H_5_	−91.797	−14.71	−22.167	36.84
DAP-1-NO_2_-2-CH_2_CH_2_CH_3_	−136.126	−70.10	−137.160	−290.82
DAP-1-NO_2_-2-OCH_3_	−117.931	−47.36	−73.976	−110.79
